# Clinical Characteristics and Outcome of Patients with Infective Endocarditis Diagnosed in a Department of Internal Medicine

**DOI:** 10.3390/jcm9030864

**Published:** 2020-03-21

**Authors:** Louis Kreitmann, David Montaigne, David Launay, Sandrine Morell-Dubois, Hélène Maillard, Marc Lambert, Eric Hachulla, Vincent Sobanski

**Affiliations:** 1 CHU Lille, Département de médecine interne et immunologie clinique, F-59000 Lille, France; 2 Centre de Référence des Maladies Autoimmunes et Systémiques Rares du Nord et Nord-Ouest de France (CeRAINO), Hôpital Claude Huriez, F-59000 Lille, France; 3 CHU Lille, Department of Clinical Physiology & Echocardiography, Heart Valve Clinic, F-59000 Lille, France; 4 Univ. Lille, Inserm U1011, Institut Pasteur de Lille, EGID, F-59000 Lille, France; 5 Univ. Lille, U1286, INFINITE, Institute for Translational Research in Inflammation, F-59000 Lille, France; 6 Inserm, U1286, F-59000 Lille, France

**Keywords:** infective endocarditis, internal medicine, echocardiography, diagnosis

## Abstract

Clinical manifestations of infective endocarditis (IE) can be highly non-specific. Our objective was to describe the clinical characteristics of patients initially referred to a department of internal medicine for a diagnostic work-up, and eventually diagnosed with IE. We retrospectively retrieved adult patients admitted to the department of internal medicine at Lille University Hospital between 2004 and 2015 who fulfilled Duke Classification criteria for definite IE. Thirty-five patients were included. The most frequently involved bacteria were non-hemolytic streptococci. Most patients presented with various systemic, cardiac, embolic, rheumatic, and immunological findings, with no sign or symptom displaying high sensitivity. The first transthoracic echocardiogram was negative in 42% of patients. Furthermore, definite diagnosis required performing at least 2 transesophageal examinations in 24% of patients. We observed a trend towards decreased survival in the subgroup of patients in whom the delay between onset of symptoms and diagnosis was >30 days. In conclusion, patients who are initially referred to internal medicine for a diagnosis work-up and who are ultimately diagnosed with IE have non-specific symptoms and a high percentage of initial normal echocardiography. Those patients require prolonged echocardiographic monitoring as a prolonged delay in diagnosis is associated with poorer outcomes such as death.

## 1. Introduction

Infective endocarditis (IE) is defined by infection of the endocardial surface of the heart, usually the valves [1,2], affecting about 5–15 per 100,000 people per year [3]. Despite significant progress in diagnosis and medical therapy, mortality rates have not changed substantially in the past 25 years, approaching 40% at one year after diagnosis [4,5,6]. The clinical presentation of IE can be highly variable and non-specific [7]. Despite prominent cardiac and septic manifestations, various signs and symptoms can be found [8,9], which are related to the immunological and embolic complications of IE, affecting the brain [10,11], skin [12,13,14], bones and joints [15,16], kidneys [17,18], and/or eyes. 

This clinical variability can cause a significant delay in the diagnosis of IE and can thus negatively affect patients’ prognosis [19,20]. While several reports have described the extra-cardiac manifestations of IE, there have been few studies, to our knowledge, focusing on patients first referred to an internal medicine department for a diagnostic work-up, in whom clinical investigations finally led to the diagnosis of IE.

In this study, we aimed to address this topic by describing the clinical, echocardiographic and microbiological characteristics in a cohort of patients diagnosed with IE in a department of internal medicine. Second, we sought to investigate the factors associated with a poor prognosis in this cohort.

## 2. Patients and Methods

### 2.1. Study Site

We conducted a retrospective monocentric cohort study of adult patients hospitalized in the department of internal medicine of Lille University Hospital (Lille, France) between January 2004 and December 2015. This 38-bed department is a tertiary referral center for systemic autoimmune diseases and the diagnostic work-up of complex clinical cases.

### 2.2. Inclusion and Exclusion Criteria

We included patients who: (a) were admitted to the department of internal medicine between 2004 and 2015; (b) received a diagnosis of IE as recorded by their attending physician in their electronic medical record; and (c) underwent ≥1 transthoracic (TTE) or transesophageal (TOE) echocardiography at the Department of Clinical Physiology and Echocardiography of Lille University hospital. We excluded patients if: (a) IE was diagnosed before the admission in the internal medicine department; (b) they had a previous medical history of systemic autoimmune or rheumatic disease; or (c) Duke classification criteria [21] were not fulfilled (after retrospective analysis of their medical chart).

### 2.3. Data Collection and Definitions

Medical charts were reviewed to collect data on demographics, comorbidities, clinical manifestations, biological and imaging characteristics of cases, treatment and follow-up. Date of IE diagnosis was defined as the date of the first echocardiography fulfilling Duke Classification major criteria for patients with positive echocardiography [21], and as the last day of hospital stay for patients with negative echocardiograms. Length of follow-up was defined as the time between date of diagnosis and either death or last alive follow-up visit. Constitutional symptoms were defined as fatigue, weight loss >10% body weight, and/or loss of appetite. Fever was defined as body temperature >38 °C. Joint and back pain was defined as subjective symptoms, whereas arthritis and bursitis were defined as objective manifestations, either clinically (tenderness, edema) or on imaging. Vascular emboli were defined either clinically (especially for digital emboli) or on imaging studies. Heart failure was defined as signs or symptoms of congestion. Anemia was defined as a hemoglobin <120 g/L in women and <130 g/L in men, thrombocytopenia as a peripheral platelet counts <100 G/L. Acute renal injury was defined as a rise in serum creatinine concentration ≥ 3 mg/L compared to baseline. Proteinuria was considered significant if ≥0.2 g/L or ≥0.3 g/day. Leukocyturia and hematuria were considered present if urine leukocytes or red cells counts were >10/μL, respectively. Recent dental care and antibiotic use were recorded as reported in the anamnestic sections of medical records, with no specified time frame. Smoking and alcohol consumption (>14 standard drinks units per week in men or >7 in women) were defined as current habits at the time of hospital admission. 

### 2.4. Data Analysis

We reported medians and interquartile ranges for quantitative variables, and percentages for categorical variables. We fitted univariate Cox proportional hazards regression models to investigate the association between clinically relevant patient characteristics and death. Multivariate regression analysis was not performed due to small sample size. We computed Kaplan–Meier estimates of survival in patients with a duration of symptoms <30 and >30 days and compared survival with the log-rank test. Analyses were conducted in the R software, using a *p*-value of < 0.05 for statistical significance. No correction was made for multiple comparisons.

### 2.5. Ethics

As per French regulatory requirements on non-interventional studies, this retrospective collection of patient data did not require written patient consent. Data confidentiality was ensured in accordance with the recommendations of the Commission Nationale Informatique et Liberté (CNIL). This study was registered in the public repository of the Institut National des Données de Santé (MR0621301219).

## 3. Results

### 3.1. Study Population

Seventy-six patients were diagnosed with IE during their hospital stay in the internal medicine department between 2004 and 2015. Out of these patients, 20 did not fulfill Duke Classification criteria for definite IE, 10 eventually received an alternate diagnosis, 9 had been diagnosed previously in another department, and 2 were previously followed for a systemic autoimmune or rheumatic disease and were subsequently excluded. Finally, 35 patients were analyzed (Supplementary Figure S1).

Baseline characteristics of patients are presented in Table 1. Patients were mainly male with a median age at diagnosis of 59 years (IQR 51–71 years). Nearly half of them had a predisposing cardiac condition, most commonly a valvular disease. None reported intravenous drug use. Four patients (11.4%) reported recent dental care. Nineteen patients (54.3%) had received antibiotics (either by their general practitioner or during a previous hospital stay).

### 3.2. Clinical, Biological and Imaging Characteristics at IE Diagnosis

Median duration of symptoms before diagnosis was 41 days (inter-quartile range 19–153 days), and it was >30 days in 20 patients (57.1%). Clinical presentation at IE diagnosis is summarized in Table 2. Twenty-six patients (74.3%) reported fever and/or constitutional symptoms. Seven patients (20.0%) presented with signs or symptoms of heart failure, and 4 patients (11.4%) with new-onset heart murmur. Eight patients (22.9%) had new-onset electrocardiogram abnormalities (atrial fibrillation, conduction or repolarization abnormalities). Nineteen patients (54.3%) suffered cardio-embolic events, most commonly affecting the fingers, lower limbs and brain. Eleven patients (31.4%) presented with objective neurological manifestations, including delirium, motor palsy and aphasia. Thirty patients underwent cerebral imaging (computed tomography (CT) or magnetic resonance imaging (MRI)), which was abnormal in 8 cases (26.7%). Rheumatic manifestations were observed in 7 patients (20%) reporting diffuse myalgia and in 10 patients (28.6%) reporting arthralgia. Arthralgias were most often diffuse (affecting shoulders, ankles, hands and wrists). Five patients (14.2%) had a clinical monoarthritis. Two patients (5.7%) had bone scintigraphy showing bilateral and symmetrical polyarthritis. Twelve patients (34.3%) had rachialgia, but only 4 of them had objective spinal lesions on imaging (2 showing sacroiliitis and 2 showing spondylodiscitis). Skin manifestations occurred in 19 patients (54.3%), including 8 with purpuric lesions (22.9%) and 3 with palmo-plantar erythematous Janeway lesions (8.6%). Seven patients (20%) had acute kidney injury. No renal biopsy was performed. 

As shown in Supplementary Table S1, median C-reactive protein level was 71 mg/L (IQR 49–141 mg/L) and median ferritin level was 324 μg/L (IQR 165–642 μg/L). Nineteen patients had abnormal immunological tests, including: rheumatoid factors (*n* = 7, 50.0%); cryoglobulinemia (*n* = 11, 50.0%), most often type III (median 0.41 g/L); hypocomplementemia (*n* = 5, 26.0%), positive antinuclear antibodies (*n* = 8, 42.0%), all of them with no specificity on immuno-blot; circulating immune complex (*n* = 5, 33%); antineutrophil cytoplasmic antibodies (ANCA) (*n* = 1, 5.0%); and antiphospholipid antibodies (*n* = 3, 2%).

### 3.3. Microbiology

Microbiological findings are presented in Table 3. Blood cultures were positive in 23 patients (65.7%), with streptococci being the most frequently isolated bacteria: group A (*n* = 1, 2.9%), group B (*n* = 1, 2.9%) and non-hemolytic streptococci (*n* = 10, 28.6%). There were 2 cases (5.7%) due to *Staphylococcus aureus* IE, 4 cases (11.4%) due to coagulase-negative staphylococci, 4 cases (11.4%) due to *Enterococcus* sp. 2 cases were polymicrobial.

Twelve patients (34.3%) had negative blood culture despite multiple testing. Microbiological diagnosis was achieved through culture of peripheral abscesses in 2 patients (5.7%) and infected valves after surgery in 1 patient (2.9%). There was one patient with *Mycoplasma pneumoniae* IE, the diagnosis of which was made because of documented seroconversion. In total, there were 8 (22.9%) cases of definite IE without any identified microorganism.

### 3.4. Echocardiography

Echocardiographic findings are presented in Table 4. Two patients had normal echocardiography. Among the 33 patients with echocardiography-proven IE, 18 (54.5%) had one vegetation, 14 (42.4%) had multiple vegetations, 7 (21.2%) had valvular perforation and 2 (6.1%) had cardiac abscesses. There were 19 (57.6%) aortic IE, 14 (42.4%) mitral IE, 2 (6.1%) tricuspid IE, and 4 patients (12.1%) had at least 2 valves involved.

Among the 33 patients with echocardiography-proven IE, the first TTE examination was conclusive for the diagnosis of IE in 19 patients (sensitivity = 57.6%). However, 14 patients (42.4%) had to undergo ≥2 TTE to identify Duke Classification echocardiographic major criteria in the diagnosis of IE. All patients underwent at least one TOE examination: either to further evaluate morphological characteristics of IE in those cases were IE diagnosis was based on a positive TTE (as per routine care at our institution); or in case of persistent clinical suspicion despite negative TTE, to increase the diagnostic yield of echocardiography. One TOE exam was sufficient to confirm IE diagnosis in 25 patients (sensitivity = 75.8%). However, 8 patients (24.3%) had to undergo ≥2 TOE before identification of echocardiographic major criteria.

### 3.5. Treatment, Follow-Up, and Survival

Median duration of follow-up was 331 days (IQR 103–1486 days). All patients received antibiotics after IE diagnosis, either based on available microbiological results or by empiric therapy. Fifteen patients (42.9%) underwent surgical valve repair. Eight patients (22.9%) patients were transferred to an intensive care unit. Eight patients (22.9%) were deceased at the end of follow-up. 

**Figure 1:**
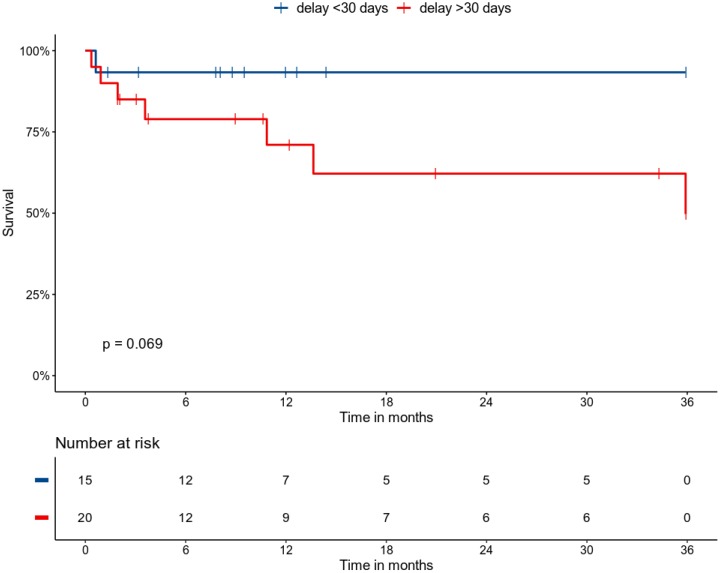
Kaplan–Meier estimates of survival in patients according to the delay between onset of symptom and accurate IE diagnosis, with between-group comparison by the log-rank test. Follow-up was right-censored at 3 years.

In univariate analysis, 3 variables were associated with patient mortality at the end of follow-up: age, immunosuppression and acute kidney injury. We observed a trend towards increased mortality in patients with duration of symptoms > 30 days before diagnosis, in patients with IE-related neurologic complications, and in patients transferred to an intensive care unit during follow-up (Table 5 and Figure 1**)**. 

## 4. Discussion

Exploring the characteristics of patients referred to our internal medicine department for a complex diagnostic work-up and eventually diagnosed with IE, we first showed that this presentation is rare, as evidenced by the small number of patients fulfilling our inclusion criteria. Our patients frequently received antibiotics before hospitalization, displayed various immunological abnormalities, as well as not systematic positive microbiology. This presentation seems challenging and prone to mis- or delayed diagnosis, with a median delay before definite diagnosis of 41 days. Importantly, a high proportion of these patients underwent valvular heart surgery, in line with the severity of this presentation.

In 2009 was published the ICE-PCS study, an international multi-center observational study describing 2781 IE cases [7]. Even though a comparison of our cohort with that of the ICE-PCS study might not lend itself to formal statistical analysis, we believe it is interesting to highlight differences in key variables assessed in both studies. Delay between first sign to admission was >30 days in 57.1% of our patients, vs. 23% in the ICE-PCS study [7]. Our patients did not present fever in 44.6% of cases, vs. 4% in [7]. Our cohort had fewer patients with classical IE predisposing conditions: no intravenous drug user (vs. 10%), 2.9% of patients with a pacemaker (vs. 10%), 2.9% of patients with a prosthetic heart valve (vs. 21%). Our patients presented more frequently with embolic events (52.8% vs. 17%), skin involvement (55.6% vs. 16%), elevated rheumatoid factors (19.4% vs. 5%). They presented less frequently with new-onset murmur (11.4% vs. 48%) and heart failure (20% vs. 32%). As it has been reported by others [3,4], *Staphylococcus aureus* was the most frequently identified bacteria in the ICE-PCS study (31%), but it accounted only for 5.6% of our cases. Overall, it appears that patients in the ICE-PCS study present with a more acute type of bacterial IE, a disease that develops suddenly and can become a life-threatening condition within a few days. On the opposite, by focusing on patients initially referred to an internal medicine department, we have selected a subgroup of IE cases for which Sir William Osler coined the term *typhoid type* IE [9,22]: a sub-acute, protracted disease due to less virulent microorganisms, with chronic immunological stimulation leading to frequent micro- and macro-embolic complications.

A key finding of our study is that no sign or symptom had high sensitivity for the diagnosis of IE in this population. The most frequent manifestations were fever and emboli, but each of these two signs were found in only <60% of patients, and up to ~25% of patients reported neither one of them. Second, if the diagnostic accuracy of transthoracic echocardiography in our cohort was in line with previous reports [21], we found that ~25% of patients with echocardiography-proven IE had to undergo at least 2 TOE examinations before final IE diagnosis. This indicates that sensitivity of one TOE examination for IE diagnosis is ~75% in our cohort, somewhat lower than the >90% reported in native- and prosthetic-valve IE in other cohorts [23]. These findings suggest that clinicians should maintain a high level of suspicion when confronted to non-specific yet compatible clinical manifestations, and that it is advisable to repeat TOE exams in case of negative findings during initial echocardiographic assessment.

We found that acute kidney injury and neurological complications of IE negatively impact patient prognosis, as it has been reported previously [11]. Interestingly, we found a trend towards decreased survival in patients with a delay between onset of symptoms and definite diagnosis of >30 days, which—to the best of our knowledge—has not yet been extensively documented. If confirmed, our finding should warrant a strong collaboration between clinicians and echocardiographists in order to maximize the chances of an accurate IE diagnosis in a timely fashion. 

Our study has several limitations. First, its retrospective design exposed to a risk of bias through incomplete collection of data. For instance, it is possible that subjective symptoms preceding IE diagnosis might have been recorded only partially in medical records. Furthermore, retrospective analysis of medical records barely allows pinpointing the clinical factors underlying the high index of suspicion that induced clinicians to repeat echocardiographic examinations. Second, the small sample size of our study precluded a more thorough statistical analysis, especially through multivariate modeling of the factors associated with a worse prognosis. We found that a longer duration of symptoms was associated with decreased survival, but this finding, however plausible, mandates caution, as our data did not allow a detailed analysis of the factors underpinning this association. Third, we restricted our analysis to patients investigated in the internal medicine department, but it would have also been interesting to study patients with a similar presentation of IE diagnosed in other departments, such as rheumatology, nephrology or dermatology units. Finally, a more detailed analysis could have been achieved in a case-control study comparing our IE patients to internal medicine patients investigated for similar non-specific symptoms, but eventually diagnosed with an authentic autoimmune disease.

## 5. Conclusions

Patients who are initially referred to internal medicine for a diagnosis work-up and who are ultimately diagnosed with IE have non-specific symptoms and a high percentage of normal echocardiography. Those patients require prolonged echocardiographic monitoring, as a delay in diagnosis is associated with a poorer prognosis reported in this manuscript.

## Figures and Tables

**Table 1:** Patients’ characteristics at baseline.

Characteristics	Number of Patients (%)
Gender (male), *n* (%)	23 (65.7%)
Age at diagnosis, median (IQR)	59 (51–71)
Diagnostic delay *, median (IQR)	41 (19–153)
Predisposing cardiac condition, *n* (%)	16 (45.7%)
Aortic valve disorder, *n* (%)	9 (25.7%)
Bicuspid aortic valve, *n* (%)	4 (11.4%)
Degenerative aortic stenosis, *n* (%)	3 (8.6%)
Aortic biologic prothesis, *n* (%)	1 (2.9%)
Mitral valve disorder, *n* (%)	5 (14.3%)
Acute rheumatic fever, *n* (%)	2 (5.7%)
Cardiac pace-maker, *n* (%)	1 (2.9%)
**Comorbid conditions**	
Cancer or hematological malignancy, *n* (%)	6 (17.1%)
Atrial fibrillation, *n* (%)	6 (17.1%)
Hypertension, *n* (%)	5 (14.3%)
Diabetes mellitus, *n* (%)	5 (14.3%)
Dyslipidemia, *n* (%)	7 (20.0%)
Current tobacco smoking, *n* (%)	9 (25.7%)
Current alcohol drinking, *n* (%)	8 (22.9%)
Intravenous drug user, *n* (%)	0 (0%)
Drug-induced immunosuppression, *n* (%)	5 (14.3%)
Recent dental care, *n* (%)	4 (11.4%)
Recent antibiotic use, *n* (%)	19 (54.3%)

* defined as duration between onset of symptoms and IE diagnosis.

**Table 2:** Clinical, biological and imaging characteristics at infective endocarditis (IE) diagnosis.

Characteristics	Number of Patients (%) ^¤^
**General manifestations**	
Fever >38 °C, *n* (%)	20 (57.1%)
Constitutional symptoms, *n* (%)	19 (54.3%)
**Neurological and ophthalmic manifestations**	
Clinical manifestations, *n* (%)	11 (31.4%)
Imaging abnormalities, *n* (%) ^#^	8 (26.7%)
Focal ischemic lesions, *n* (%)	4 (13.3%)
Multiple ischemic lesions, *n* (%)	2 (6.7%)
Subarachnoid hemorrhage, *n* (%)	2 (6.7%)
Intraparenchymal hemorrhage, *n* (%)	2 (6.7%)
Cerebral vasculitis, *n* (%)	1 (3.3%)
Optic fundus abnormalities, *n* (%) ^$^	2 (16.6%)
**Cardiac manifestations**	
Heart failure, *n* (%)	7 (20.0%)
Murmur, *n* (%)	22 (62.9%)
New-onset murmur, *n* (%)	4 (11.4%)
New-onset EKG abnormalities, *n* (%)	8 (22.9%)
**Embolic manifestations**	
Any embolic manifestation, *n* (%)	19 (54.3%)
Digital emboli, *n* (%)	7 (20.0%)
Lower limb emboli, *n* (%)	4 (11.4%)
Cerebral emboli, *n* (%)	5 (14.3%)
Renal emboli, *n* (%)	3 (8.6%)
Splenic emboli, *n* (%)	2 (5.7%)
Bone emboli, *n* (%)	2 (5.7%)
**Rheumatic manifestations**	
Myalgia, *n* (%)	7 (20.0%)
Arthralgia, *n* (%)	10 (28.6%)
Rachialgia, *n* (%)	12 (34.3%)
Peripheral arthritis, *n* (%)	5 (14.3%)
**Skin manifestations**	
All manifestations, *n* (%)	19 (54.3%)
Purpura, *n* (%)	8 (22.9%)
Janeway palmo-plantar lesions, *n* (%)	3 (8.6%)
Skin biopsy showing vasculitis, *n* (%) *	7 (87.5%)
**Hematological abnormalities**	
Polyadenopathy, *n* (%)	5 (14.3%)
Splenomegaly, *n* (%)	1 (2.9%)
Anemia, *n* (%)	27 (77.1%)
Leukocytosis, *n* (%)	11 (31.4%)
Thrombopenia, *n* (%)	4 (11.4%)
**Renal manifestations**	
Acute kidney injury, *n* (%)	7 (20.0%)
Proteinuria, *n* (%)	7 (20.0%)
Leukocyturia/hematuria, *n* (%)	5 (14.3%)

**^¤^** Unless specified, number of patients affected and related percentages are reported over the whole cohort (*n* = 35). ^#^ 30 over 35 patients underwent cranial imaging (CT-scan and/or MRI), ^$^ 12 over 35 patients underwent optic fundus examination, and * 8 over 35 patients had skin biopsy. CT: computed tomography; MRI: magnetic resonance imaging; EKG: electrocardiogram.

**Table 3:** Microbiology results.

Characteristics	Number of Patients (%)
IE with positive blood cultures, *n* (%)	23 (65.7%)
IE with negative blood cultures, *n* (%)	12 (34.3%)
IE with negative microbiology, *n* (%)	8 (22.9%)
**Identified bacteria**	
*Streptococcus* sp., *n* (%)	12 (34.3%)
*Streptococcus pyogenes*, *n* (%)	1 (2.9%)
*Streptococcus agalactiae*, *n* (%)	1 (2.9%)
Non-haemolytic streptococci, *n* (%)	10 (28.6%)
*Staphylococcus* sp., *n* (%)	9 (25.7%)
*Staphylococcus aureus*, *n* (%)	2 (5.9%)
Negative coagulase staphylococci, *n* (%)	7 (20.0%)
*Enterococcus* sp., *n* (%)	5 (14.3%)
*Enterococcus faecalis*, *n* (%)	3 (8.6%)
*Enterococcus faecium*, *n* (%)	1 (2.9%)

IE with negative microbiology refers to cases where no bacteria could be identified despite repeated blood cultures, sampling of peripheral foci of infection and indirect microbiological identification methods (such as serology). IE with negative blood cultures refers to cases with negative blood cultures but successful bacterial identification through other means. Non-haemolytic streptococci belonged to the following species: *Streptococcus oralis*, *Streptococcus mutans*, *Streptococcus gallolyticus*, *Streptococcus agalactiae*, *Streptococcus homans*, *Streptococcus bovis*, *Streptococcus parasanguinis*. Negative coagulase staphylococci belonged to the following species: *Staphylococcus epidermidis*, *Staphylococcus hominis* and *Staphylococcus warneri*.

**Table 4:** Echocardiographic findings.

Characteristics	Number of Patients (%)
**Valves involved**	
Aortic valve, *n* (%)	19 (57.6%)
Mitral valve, *n* (%)	14 (42.4%)
Tricuspid valve, *n* (%)	2 (6.1%)
Mono-valvular involvement, *n* (%)	28 (84.8%)
Bi-valvular involvement, *n* (%)	4 (12.1%)
Pacemaker, *n* (%)	1 (3.0%)
**Nature of lesions**	
1 vegetation, *n* (%)	18 (54.5%)
≥2 vegetations, *n* (%)	14 (42.4%)
Valvular destruction, *n* (%)	7 (21.2%)
Abscess, *n* (%)	2 (6.1%)
**Number of TTE examination before definite diagnosis**	
1 TTE, *n* (%)	19 (57.6%)
2 TTE, *n* (%)	8 (24.2%)
3 TTE, *n* (%)	6 (18.2%)
**Number of TOE examination before definite diagnosis**	
1 TOE, *n* (%)	25 (75.8%)
2 TOE, *n* (%)	6 (18.2%)
3 TOE, *n* (%)	2 (6.1%)

Percentages are reported over patients with positive echocardiography (*n* = 33). TTE: transthoracic echocardiography; TOE: transesophageal echocardiography.

**Table 5:** Factors associated with death by univariate Cox proportional hazards regression modelling.

Variables	HR	CI_95%_	*p*-Value
Gender (male)	0.53	0.13–2.18	0.38
Age (per year)	1.07	1.01–1.14	0.03
Current smoking	1.34	0.33–5.44	0.69
Current alcohol drinking	0.55	0.11–2.61	0.46
Immunosuppression	4.1	1.32–12.72	0.02
Duration of symptoms > 30 days	5.6	0.66–47.11	0.11
Predisposing condition	1.87	0.47–7.46	0.37
Fever	0.71	0.18–2.73	0.61
Constitutional symptoms	1.59	0.42–6.02	0.5
Neurological symptoms	3.49	0.79–15.48	0.1
Acute kidney injury	4.5	1.13–17.88	0.03
Embolic manifestations	1.11	0.24–3.34	0.88
Heart failure	2.67	0.68–10.54	0.16
Skin manifestations	0.8	0.22–2.92	0.73
C-reactive protein (mg/L)	1	0.995–1.005	0.87
Immunological manifestations	1.5	0.39–5.86	0.56
Transfer to ICU	3.38	0.85–13.55	0.09

Duration of symptoms is defined as the time in days between onset of symptoms and IE diagnosis; predisposing condition refers to any heart or valve disease known to be a risk factor of IE; constitutional symptoms is defined as fatigue, weight loss > 10% body weight, and/or loss of appetite; acute cardiac manifestations is defined as signs or symptoms of heart failure, new-onset murmur and/or new-onset EKG changes; immunological manifestations is defined as any abnormality between rheumatoid factors, cryoglobulinemia, hypocomplementemia, antineutrophil cytoplasmic antibodies, antinuclear antibodies, circulating immune complexes and antiphospholipid antibodies. HR: hazard-ratio; CI_95%_: 95% confidence interval; ICU: intensive care unit.

## Supplementary Materials

The following are available online at https://www.mdpi.com/2077-0383/9/3/864/s1, Figure S1: Flow-chart of the study, Table S1: Selected biological parameters at IE diagnosis
