# Supplementary material for: Clinical Characteristics and Outcome of Patients with Infective Endocarditis Diagnosed in a Department of Internal Medicine
Source: J Clin Med. 2020 Mar 21;9(3):864. doi: 10.3390/jcm9030864 (PMC7141516; doi:10.3390/jcm9030864)
Supplement: Supplementary file 1 [file jcm-09-00864-s001.pdf]

Supplementary Materials: Clinical Characteristics and Outcome of Patients with Infective Endocarditis Diagnosed in a Department of Internal Medicine

Louis Kreitmann, David Montaigne, David Launay, Sandrine Morell-Dubois, Hélène Maillard, Marc Lambert, Eric Hachulla and Vincent Sobanski

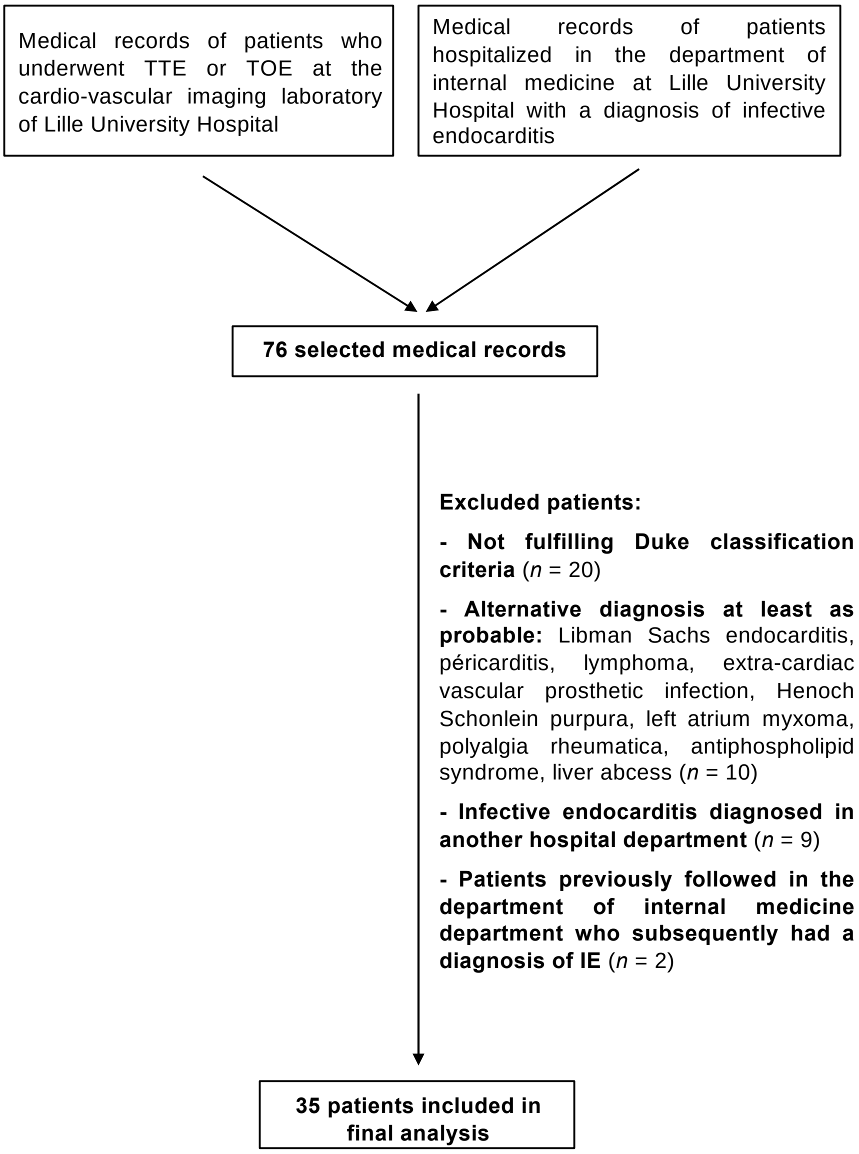

Figure S1. Flow-chart of the study.

Table S1. Selected biological parameters at IE diagnosis.

**Parameters**	**Patients tested**	**Patients affected**
C-reactive protein (mg/L), median (IQR)	34	71 (46–144)
Ferritin (µg/L), g/L), median (IQR)	18	324 (165–642)
Rheumatoid factors, *n* (%)	14	7 (50.0%)
Cryoglobulinemia, *n* (%)	22	11 (50.0%)
Hypocomplementemia, *n* (%)	19	5 (26.3%)
ANCA, *n* (%)	20	1 (5.0%)
Anti-nuclear antibodies, *n* (%)	19	8 (42.1%)
Circulating immune complexes, *n* (%)	15	5 (33.3%)
Antiphospholipid antibodies, *n* (%)	14	3 (21.4%)

ANCA: antineutrophil cytoplasmic antibodies. Of note, the patient with positive ANCA had positive immunofluorescence but negative enzyme-linked immunoassay for both PR proteinase 3 (PR3) and myeloperoxidase (MPO).
